# Endocytosis: The Nanoparticle and Submicron Nanocompounds Gateway into the Cell

**DOI:** 10.3390/pharmaceutics12040371

**Published:** 2020-04-17

**Authors:** Darío Manzanares, Valentín Ceña

**Affiliations:** 1 Unidad Asociada Neurodeath, Universidad de Castilla-La Mancha, 02006 Albacete, Spain; dario.manzanares@uclm.es; 2 CIBERNED, Instituto de Salud Carlos III, 28031 Madrid, Spain

**Keywords:** nanoparticles, endocytosis, clathrin, caveolin, macropinocytosis

## Abstract

Nanoparticles (NPs) and submicron particles are increasingly used as carriers for delivering therapeutic compounds to cells. Their entry into the cell represents the initial step in this delivery process, being most of the nanoparticles taken up by endocytosis, although other mechanisms can contribute to the uptake. To increase the delivery efficiency of therapeutic compounds by NPs and submicron particles is very relevant to understand the mechanisms involved in the uptake process. This review covers the proposed pathways involved in the cellular uptake of different NPs and submicron particles types as well as the role that some of the physicochemical nanoparticle characteristics play in the uptake pathway preferentially used by the nanoparticles to gain access and deliver their cargo inside the cell.

## 1. Introduction

Nanomedicine has become one of the most rapidly growing areas of research in the biomedical field during the last years. Nanoparticles (NPs) and submicron particles (named both from this point as NPs to abbreviate) are defined as materials with nanometric sizes (1–100 and 100–1000 nm, respectively) that interact with biological systems in an unusual way because of their high surface to volume ratio. This property, combined with the possibility of modifying their peripheral chemical groups to achieve multitasking properties, provides NPs compounds with a very high potential for diagnostic and therapeutic applications in nanomedicine. Thus, they offer the potential for a more selective and accurate treatment in a huge variety of pathologies including infectious, auto-immune and inflammatory processes, cancer or neurodegenerative diseases, among many others, by acting as carriers of drugs enabling a targeted delivery of therapeutic agents (from small drugs to genetic material) at the cellular or subcellular level, or by the therapeutic properties of the NP itself. In addition, NPs have also potential usefulness in diagnostics which might lead to the improvement of many of currently performed medical procedures [1,2].

The initial interaction between NPs and their cargo with the target cell involves uptake into the cell, being the internalization pathway very relevant to achieve the intended effect. Nanoparticles can gain access to the cell interior through simple diffusion or translocation, an energy-independent process that depends on the NP concentration gradient, but also on other factors such as its liposolubility [1]. However, the most common mechanism used by NPs to enter the cells is an energy-dependent process named endocytosis that has been described as the uptake of substances from the extracellular environment by vesicles generated from the cell plasma membrane [3].

The knowledge of the uptake route is important because, depending on it, the fate of the NP can be different, being useful or not for certain purposes. For instance, transcytosis (in which a vesicle travels across a cell) is very important in certain processes such as the gastrointestinal absorption for oral preparations or to be incorporated into the blood stream. In this case, caveolin-mediated endocytosis (CVME) plays the main role. In addition, many therapeutic compounds can be designed to arrive to specific cellular organelles where they can play a therapeutic role, being the uptake pathway determinant in their intracellular fate. This is the case for NPs taken up by CVME, which involves endoplasmic reticulum and Golgi complex, and could be useful to target those organelles and to carry certain drugs there. In addition, the uptake route can also lead to less endosomal degradation and larger cargo release to the cytosol depending on the NP employed and the cell type. Thus, CVME seems to avoid the endo-lysosomal system in some cell types while other authors report that macropinosomes are more likely to liberate their content without lysosomal degradation [4].

In addition, NPs properties can have a great influence in the endosomal escape before fusing lysosomes. So, there are several mechanisms by which NPs can escape the endosomes including: (a) proton sponge effect which involves un-protonated amine groups of NPs absorbing protons due to endosome acidification, triggering the entrance of Cl- ions and, consequently, water by osmosis, causing the endosome rupture, (b) umbrella effect which involves amine protonation leading to charge repulsions, which would expand the structure leading to endosomal rupture, (c) direct fusion of NPs with the endosome membrane and formation of pores in the endosome surface due to the induction of membrane stress and internal membrane tension [5].

This article presents an overview of the pathways by which the different types of NPs gain access to the cell interior.

## 2. Classification of the Endocytic Pathways

**Figure 1:**
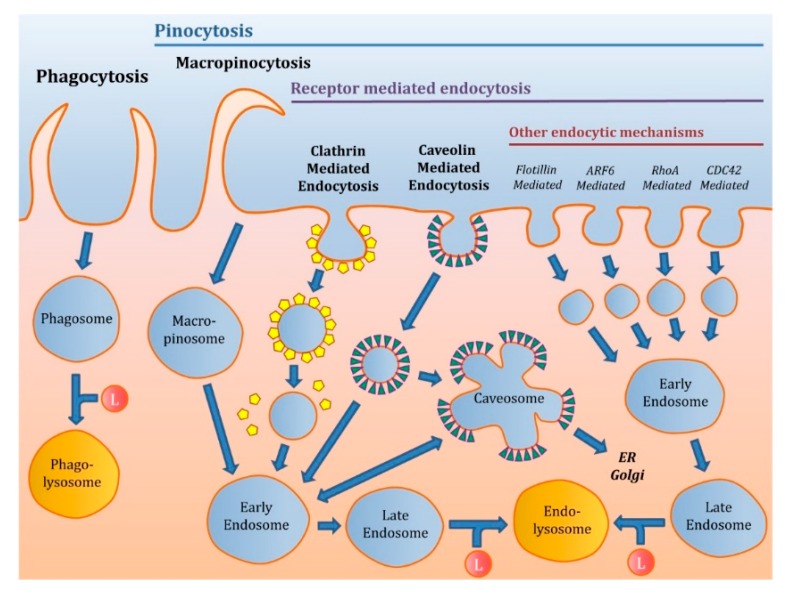
Main energy-dependent uptake pathways of the cell. Macropinocytosis forms macropinosomes that could finally join the early endosomes. Clathrin-mediated endocytosis (CME) and caveolin mediated endocytosis (CVME) are the main receptor-mediated endocytosis (RME) processes. On the other hand, other endocytic RME mechanisms as flotillin, ARF6, RhoA, or CDC42 mediated endocytosis are also present in the cell. The final fate of endosome vesicles is to fuse with lysosomes.

There are different mechanisms of endocytosis (Figure 1) that are generally classified as follows:

### 2.1. Phagocytosis

Phagocytosis consists in the uptake by the cell of opsonized particulate substances and solutes by vesicles with a size in the micrometer range that incorporate large plasma membrane surface areas [3]. Since phagocytosis is barely used as a mechanism for NPs uptake, it will not be described in detail in this review.

### 2.2. Pinocytosis

Pinocytosis involves the uptake of fluids containing solutes and particles by vesicles of smaller size than those generated during phagocytosis. This endocytic mechanism can be classified in macropinocytosis and receptor-mediated endocytosis (RME).

#### 2.2.1. Macropinocytosis

Macropinocytosis allows the uptake of material through large vacuoles, variable in size, called macropinosomes. After internalization of macropinosomes, pH decreases and endosome markers start to appear. Later, the acidified macropinosomes can either fuse with late endosomes, with lysosomes or recycle their cargo to the membrane [6].

#### 2.2.2. Receptor-Mediated Endocytosis (RME)

**Figure 2:**
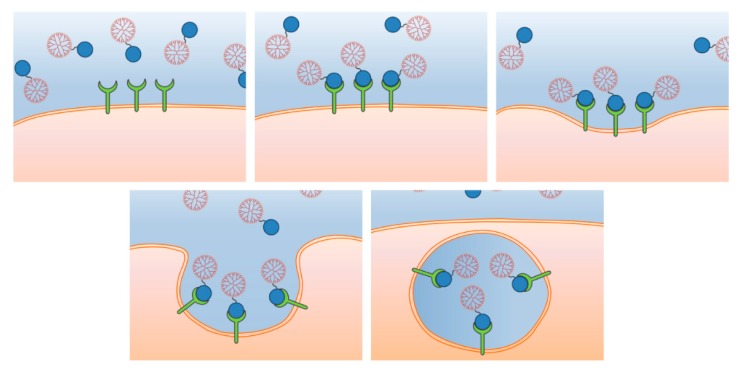
Receptor mediated endocytosis. A specific interaction of a ligand on the surface of a nanoparticle (NP) (in blue) with a receptor (in green) triggers the formation of an invagination on the cell membrane which finally leads to the formation of an endosome thanks to the proteins implicated in each of the different pathways.

Receptor-mediated endocytosis represents the most common pathway followed by NPs to get access to the cell interior. It starts by the binding of a ligand, attached to the NP, to a specific receptor that triggers a conformational change leading to an invagination of the plasma membrane that generates an early endosome (Figure 2). There are different types of RME, being the more relevant ones for NP uptake the following:

##### Clathrin-Mediated Endocytosis (CME)

Clathrin-mediated endocytosis (CME) takes place in specialized plasma membrane regions where clathrin is recruited. Formation of endocytic clathrin-coated vesicles, with a size range of 70–150 nm depending on the cell type [7], is triggered by the interaction of an agonist with its receptor which leads to the assembly of clathrin into a polygonal form, coating the vesicle. Then, the vesicle internalizes, loses its clathrin coat, and fuses with other vesicles to form an early endosome that turns into a late endosome that fuses with a lysosome [8].

##### Caveolin-Mediated Endocytosis (CVME)

Caveolin mediated endocytosis consists of invaginations of 60 to 80 nm of the plasma membrane that can take up extracellular fluid content. The proteins that are involved in this endocytic pathway, like caveolin-1, bind to cholesterol in lipid rafts and they do not dissociate from the vesicles after the uptake, unlike it happens during CME. Caveolin vesicles are formed and fuse with other caveolin vesicles, leading to multicaveolar structures called caveosomes that fuse with early endosomes in a bidirectional way. From this point, the vesicular structures can travel to the smooth endoplasmic reticulum or to the Golgi-trans network depending on the cell type [9].

##### Other Pathways

In addition to the mechanisms described above, several clathrin- and caveolin-independent pathways exist such as Arf-6, Rho-A (or IL2Rb-dependent pathway), flotillin, or CDC42 (CLIC/GEEC)-dependent endocytosis, but these pathways will not be discussed further in the present review, since they do not contribute significantly to cellular NP uptake.

## 3. Methodology to Elucidate the Different Endocytosis Pathways of Nanoparticles (NPs) in Cells

The range of energy dependent pathways by which a NP can enter the cell is very wide and requires a study of the concrete routes that could be implicated in the uptake. For that purpose, concrete endocytic pathways are blocked and changes in the entrance of NPs are observed by different techniques such as fluorescent microscopy or flow cytometry. Traditionally, the study of the influence of different endocytosis pathways has been carried out by a pharmacologic approach, which consists of the employment of different chemical inhibitors to block several endocytosis pathways. Thus, for inhibiting CME treatments such as chlorpromazine (which acts through a reversible translocation of clathrin and its adapter proteins, from the cellular membrane to intracellular vesicles) are used. To block CVME, compounds as methyl-β-cyclodextrin (that removes cholesterol out of the plasma membrane, inhibiting thus cholesterol-dependent mechanisms as CVME) or genistein (that blocks the recruitment of dynamin II and perturbs the actin network, which are fundamental processes in CVME) [10] are employed and for macropinocytosis treatments such as amiloride (which inhibits Na+/H+ exchange, a process that affects macropinocytosis by lowering the pH in the submembranous region) [11] are used.

In addition, there are other new approaches such as gene silencing of key proteins for each pathway. However, this method is not yet widely employed and most of evidence available nowadays is based on the pharmacologic approach.

## 4. Influence of NP Physical Properties on the Cellular Uptake

It is known that there are several factors that can strongly influence the uptake of a NP, being able to change the endocytosis pathway and their intracellular fate [12]. The most important ones are NP size, charge, shape, and rigidity.

### 4.1. Size

It is generally accepted that NP internalization into non-phagocytic cells is larger for smaller particles [13] being the optimal size for effective uptake near to 50 nm, dependent of the type of NP as it happens for gold NPs [14]. Furthermore, it is difficult to establish a pattern of size and endocytic pathway because particles can interact with specific receptors that trigger one pathway or another and can form clusters on the surface that increment the overall size. However, it has been reported that NPs sizes up to 150 nm are mostly internalized via CME or CVME with a maximum size of 200 nm, while 250 nm to 3 μm ones have shown to have an optimal in vitro uptake by macropinocytosis and phagocytosis [15].

### 4.2. Charge

Charge plays a relevant role in NPs uptake. Thus, cationic NPs are better internalized into the cells due to the cell surface negative charges while, neutrally or negatively charged NPs are less efficiently internalized by the different cells [16]. Moreover, charge also influences the uptake pathway, being negatively charged NPs more easily taken up into cells by CVME [17] while positively charged NPs seem to prefer CME [16].

### 4.3. Shape

There is no general agreement on whether the NP shape (sphere-like, cylinders, ellipses, rods, or disks) may influence both the extent of the uptake and the endocytosis pathway. So, it has been proposed that spherical NPs such as gold or PEGylated NPs have a higher uptake rate [18] while other authors propose that elongated NPs are better endocytosed than the spherical ones [19]. The reasons for these discrepancies are not clear, but the different types of cells used in those studies might contribute to it.

### 4.4. Rigidity

Nanoparticle rigidity seems to increase endocytosis in comparison to soft NPs. Furthermore, rigid NPs are more likely to be taken up by CME while more flexible NPs are endocytosed by macropinocytosis [20].

### 4.5. Other Factors

In addition to the parameters described above, there are others that can influence the rate of NPs uptake such as the interaction with serum proteins or the lipophilicity. Moreover, interactions of the NPs with serum proteins can trigger the formation of a protein corona over the NP surface leading to an increase in size which might affect the interaction between the NP and the cell [21]. On the other hand, lipophilic NPs might enter the cells by passive diffusion, by directly interacting with the lipidic part of the cell membrane, [22]. These factors can be considered (among others) as critical design parameters to be taken into account in order to synthetize more efficient NPs [23].

## 5. Endocytosis Pathways for Nanoparticles

**Figure 3:**
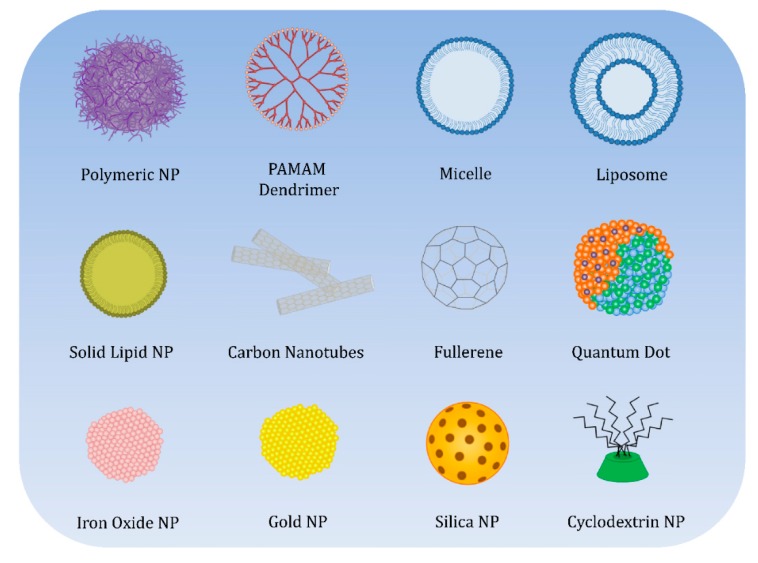
Representation of the different kinds of NPs included in this review.

Once the main mechanisms and factors that can have an influence on endocytosis have been described, it is important to make an assessment of the uptake followed by the different types of NPs kind by kind, classified by their nature, including polymeric NPs (natural and synthetic), dendrimers, lipidic NPs, carbon based NPs, quantum dots (QDs), metallic NPs, mesoporous silica NPs (MSNs), β-cyclodextrin based NPs (CDNPs), and micelles. A scheme of the different NPs discussed in the review can be found in Figure 3.

### 5.1. Polymeric NPs

#### 5.1.1. Natural Polymers

##### Chitosan Submicron NPs (CSNPs)

Chitosan [(1, 4)-2-amino-2-deoxy-d-glucan] (CS) is a linear polyaminosaccharide that is obtained by N-deacetylation of chitin [24]. Most of the chitosan submicron NPs (CSNPs) mainly enter the cells by CME, independently of their size, as happens for 250 nm sized ones in macrophage murine cell line RAW 264.7 [25] or 15.6 ± 3.5 nm sized in Caenorhabditis elegans [26]. However, others with a size close to or below 200 nm can enter the cells by macropinocytosis and CVME, this one to a lesser extent in human cervical carcinoma HeLa cells [27]. In addition, chemical modifications of this kind of nanoparticles might not have influence on the main CME pathway, as happens for cholesterol modified CSNPs [28]. However, other chemical changes in CS-based NPs might modify the pathway for cellular uptake. So, while unmodified CSNPs enter the cells mainly by CME and macropinocytosis (with a secondary intervention of CVME), the addition of polyethylene glycol (PEG) makes macropinocytosis play a main role, probably due to an increase in size [27].

##### Albumin-Based NPs

Albumin-based based NPs are widely employed due to properties such as high-water solubility and biocompatibility, and low toxicity and immunogenicity. Furthermore, these NPs have got many carboxylic and amino groups that can be used as binding sites for drugs [29]. These NPs enter the cells mainly by CME, independently of their charge. That is the case for electronegative bovine serum albumin (BSA) 150 nm sized NPs loaded with gemcitabine in MIA PaCa-2 and PANC-1 human pancreatic carcinoma cell lines [30]. CME is also the main uptake pathway for electropositive human serum albumin (HSA)-based NPs loaded with lapatinib in SK-BR-3 human breast adenocarcinoma cells, having an approximated size of 140 nm [31] and in modified albumin NPs as galactosylated curcumin-loaded BSA NPs (Gal-BSA-Cur) in human colon adenocarcinoma Caco-2 cells [29]. On the other hand, for electronegative plasmid loaded has-based NPs, having a size of 120 nm, the uptake is carried out mainly by CVME in cultured human retinal pigment epithelial (ARPE-19) cells [32].

##### Alginate NPs

Alginate is an anionic natural polymer employed in biomedical applications due to its high biocompatibility and low toxicity. Uptake of alginate-based NPs is highly dependent on the size. Thus, oleoyl alginate ester NPs of 50 and 120 nm in size enter the cells by CME, 420 nm sized ones do it by CVME and 730 nm sized NPs are taken up by macropinocytosis in human colon adenocarcinoma Caco-2 cells [33].

#### 5.1.2. Synthetic Polymers

##### Polystyrene NPs

Polystyrene is a biocompatible and hardly biodegradable aromatic polymer formed by the polymerization of styrene monomers. Polystyrene NPs generally enter the cells by CME [23], even when they have quite different sizes (44 and 100 nm) [34]. Furthermore, the uptake seems to vary depending on the cell type as well. Thus, in macrophages, the uptake is carried out by CME and phagocytosis, while for human lung carcinoma A549 cells depend on CME and CVME [35]. Other authors have even described the uptake mechanism into BOEC bovine oviductal epithelial cells and HCF human colon fibroblasts through passive translocation [36].

##### Poly(lactic-*co*-glycolic) (PLGA) NPs

Poly (lactic-co-glycolic acid) is a widely used biodegradable NP that is easily metabolized being converted to lactic and glycolic acids. It is generally assumed that PLGA NPs are taken up by CME mainly if they are positively charged, as happens in L5178Y mouse and TK6 human lymphoblasts, while for negative charged ones the entrance is weak and CME and CVME independent [37,38]. Nevertheless, NP decoration with CS alters the uptake pathway, being able to enter the cells by macropinocytosis as well, apart from CME in human colon adenocarcinoma Caco-2 cells [39]. Some other modifications such as it happens with CSKSSDYQC-dextran-poly(lactic-co-glycolic acid), can make the NPs enter the cells not only by CME, but also by CVME in human colon adenocarcinoma Caco-2/mucus secreting human colon HT-29-MTX cocultured cells [40]. However, other chemical modifications such as addition of PEG do not seem to alter the CME pathway in rat glomerular mesangial cells (HBZY-1) [41]. That is also the case for PEGylated PLGA NPs loaded with Zinc phthalocyanine and Zinc naphthalocyanine in human breast adenocarcinoma MCF-7 cells [42].

##### Polyethylenimine (PEI) NPs

Polyethylenimine is a synthetic, aliphatic, and slightly basic polycationic polymer which is formed by the polymerization of aziridine and can be linear or branched [43]. For this kind of nanoparticle, CME is the main endocytic pathway involved in cellular uptake, as it happens in rat neural cells [44]. However, other authors consider that CVME is also involved, as PEI NPs is almost equally taken up by CME and CVME pathways in A549 human lung carcinoma and HeLa human cervix carcinoma cells [45,46]. In fact, several data reveal that polyethylenimine (PEI) branched nanoparticles could show some preference for cholesterol dependent pathways like CVME, while linear PEI is preferentially taken up by the CME pathway [43]. As widely mentioned in this review article, NP chemical modifications can influence the preferential pathway for cellular PEI uptake. Thus, Asn-Gly-Arg (NGR) peptide-modified multifunctional poly(ethyleneimine)-poly(ethylene glycol)-based NPs internalize via CVME [47] in human umbilical vein endothelial cells (HUVEC) while PEI incorporating a lipid coat decorated with apolipoprotein A-1 enter the cells by CME in RAW 267.4 macrophage murine cell line [48]. However, adsorption of different proteins or serum such as albumin, fetal bovine serum (FBS), fibronectin, or collagen I (among others) onto PEI NPs can influence the uptake [49]. Under these circumstances, CVME seems to play a major role in NP internalization, being this larger when fibronectin or collagen I are adsorbed onto the NP [49]. On the other hand, bile acid-PEI NPs can enter the cells through direct translocation due to the capability of bile acids of interacting with the cellular membrane and promote the uptake of polar molecules [50].

### 5.2. Dendrimers

Dendrimers are polymer molecules containing cascades of repeated branches grown from one or several cores. They contain three architectural domains: (i) the core, to which the branches are attached, (ii) the shell of the branches surrounding the core, and (iii) the multivalent surface formed by the branches’ termini. Most of the dendrimer internalization studies have been performed using PAMAM since this dendrimer has been the most widely used in biological experiments. PAMAM dendrimers are endocytosed by an energy-dependent process and its uptake depends on the cell type and the dendrimer generation. The highest internalization rate for PAMAM dendrimers is observed with G4, followed by G3 and G2 [43]. Charge also plays an important role in the selection of the endocytic pathway used by dendrimers. Thus, G4 PAMAM cationic dendrimers, generally –NH2 terminated, are preferentially endocytosed by CME, as it happens in mouse hippocampal neurons [51]. However, in certain cell types like human breast adenocarcinoma MCF7 cells, PAMAM G4 NH2-terminated can be endocytosed by macropinocytosis in addition to CME [52]. Furthermore, PAMAM G4 dendrimers, both cationic and neutral, seem to be internalized by a caveolin- and clathrin-independent pathway in human lung carcinoma A549 cells [53] while low generation (G2) amine-terminated dendrimers are also taken up mainly by CME pathway in human colon adenocarcinoma Caco-2 cells [54] and by CME and CVME in HEK293 human embryonic kidney epithelial cells [53]. On the other hand, anionic, -COOH and –OH terminated dendrimers, are taken up by CVME [41] even though some researchers suggest that CME could also play a role [55].

As it happens with the rest of NPs, chemical modification can have an influence on the uptake pathway. Thus, for generation 4.5 PAMAM dendrimers, their usual CME endocytotic pathway switches to CVME when these particles are PEGylated in human pharynx squamous cell carcinoma (FaDu) cells [56]. This is also the case for chondrocyte affinity peptide modified PAMAM conjugate, which pathway depends on CME as well as CVME in rat chondrocytes [57]. Furthermore, for PAMAM G4 with amine groups in a 75% and folate ones in a 25% are taken up by both CME and CVME while the ones with a 25% of acrylate group and 50% of PEG do not enter mouse hippocampal neurons [51].

### 5.3. Lipidic NPs

#### 5.3.1. Liposomes

Liposomes are spherical particles with an average size in the range of 100–150 nm with walls composed by a single or various lipid bilayers, containing an hydrophilic cavity, being thus able to transport cargos with different physical properties related to their polarity [58]. It would be expected that the lipidic nature of liposomes would facilitate entry into the cell by plasma membrane translocation. However, most liposomes enter the cells by CME, as is the case for UROtsa human urothelium bladder cells, A431 human epidermoid cancer cells [59] and Hep-2 human hepatocarinoma cells, in the latter case CVME and macropinocytosis also participate in the NP uptake, but at a minor degree [60]. Cell type, liposome lipid composition and ligand decoration can switch the preferential way for cell entry from one pathway to another. Thus, liposomes modified with octaarginines and cholesterol enter into mouse colon carcinoma C26 cells by both CME and macropinocytosis, which is also the case in A549 human lung carcinoma cells, with a main role for CME and the intervention of CVME as well [61]. Furthermore, liposomes decorated with fusogenic peptides and lipids such as the zwitterionic lipid dioleoylphosphatidylethanolamine can follow lipid-rafts mediated endocytosis related to CVME as described for human hepatocyte carcinoma Hep G2 and human malignant melanoma A375 cells [62]. However, other modifications do not seem to change the CME pathway that liposomes usually follow, as it happens with GALA peptide (WEAALAEALAEALAEHLAEALAEALEALAA)-modified liposomes in human lung microvascular endothelial cells (HMVEC-L) [63] or liposomes containing a malachite green derivative in the lipid membrane [64] in mouse colon adenocarcinoma Colon 26 cells. Nevertheless, the entrance of lipid modified liposomes depends also on the cell type, being this the case for exosome-mimicking liposomes that were formulated with DOPC/SM/Chol/DOPS/DOPE, where the uptake is dependent on CVME and macropinocytosis in A549 cells, while for human umbilical vein endothelial cells (HUVEC) it depends on CME [65]. Liposome charge also plays a relevant role in selecting the endocytosis pathway. So, in U87-MG human glioblastoma cells, charged liposomes (about 100 nm), both cationic and anionic, were taken up mainly by macropinocytosis while neutral ones were more likely taken up by CVME. However, as mentioned before, liposomes uptake also seems to be cell-dependent since, in NIH/3T3 mouse fibroblast cells, the three kinds of liposomes mentioned above tend to enter to the cell by CME [66].

#### 5.3.2. Solid Lipid NPs (SLNs)

Solid Lipid Nanoparticles (SLNs) are colloidal particles composed by a lipid matrix that is solid at room and physiological body temperature. These particles are biocompatible, biodegradable, and provide a controlled drug delivery. When bound to DNA, they enter the cell by CME, as it happens in HEK293T human embryonic kidney epithelial cells [67]. Nevertheless, several other pathways, like CVME and other dynamin-dependent processes, can be involved in SLNs uptake by human colon adenocarcinoma Caco-2 cells [68]. Once more, modifications of the NP lead to preferential entry pathways. So, addition of protamine-dextran-DNA leads the SLNs to be taken up mainly by macropinocytosis. However, stabilization of SLNs with either polysorbate 60 or 80 leads the nanoparticle to a CME pathway in four human glioma cell lines (A172, U251, U373, and U87) [69]. The same happens for apolipoprotein E-functionalized SLNs [70].

### 5.4. Carbon Based Nanoparticles

Due to several unique properties, as biocompatibility or mechanical strength, carbon NPs are widely used as drug delivery systems [71]. Within this group of molecules, there is a huge variety of structures and combinations. This review will only cover endocytosis pathways followed by carbon nanotubes, fullerenes, and carbon oxide derivatives.

#### 5.4.1. Carbon Nanotubes

##### Single-Walled Carbon Nanotubes (SWCNTs)

Single wall carbon nanotubes have a cylindrical shape and possess unique mechanical, electrical, and optical properties. Apart from pinocytosis, in which macropinocytosis plays the most important role (followed by CVME and CME) for long (630 ± 191 nm), medium (390 ± 50 nm), and short length (195 ± 63 nm) nanotubes [72], uptake of single-walled carbon nanotubes (SWCNTs) involves non-specific interactions with the cell membrane which changes the membrane tension to favor endocytosis [73]. On the other hand, very short SWCNTs (50 nm or smaller), are capable to enter the cell by direct insertion and diffusion through the cell membrane [74].

##### Multi-Walled Carbon Nanotubes (MWCNTs)

Multi-walled carbon nanotubes (MWCNTs) have a structure based on two or more concentric carbon nanotubes. They have higher mechanical strength and thermal stability than SWCNTs. MWCNTs entry into the cell seems to depend on both CME and CVME, as it happens in human lung epithelial BEAS-2B cells [75]. As for other NPs, chemical modification can have an influence on the uptake pathway. Thus, for instance, decoration of MWCNTs with recombinant ricin A-chain leads the NP to be taken up mainly by CME in human cervical carcinoma HeLa cells [76].

#### 5.4.2. Fullerenes

Fullerenes are defined as aromatic carbon-based compounds that form a spherical and closed structure that is defined by the number of carbons composing it [77]. Unmodified C60 fullerenes (about 1 nm size) can enter RAW 264.7 immortalized murine macrophages by passive diffusion [78]. However, once they are modified, cell uptake is achieved through an energy-dependent process. Thus, C60 fullerenes coupled to phenylalanine/poly-lysine derivatives enter the cell through caveolin-lipid rafts in HEK293 human embryonic kidney epithelial cells while they are taken up mainly by CME in 3T3 L1 rat fibroblast and RH-35 rat hepatoma cells [79].

#### 5.4.3. Carbon Oxide NPs

These negatively charged NPs are prepared from graphite and are stable and water dispersible, being also able to be used as carriers for molecular cargos. These NPs have been also called “membrane penetrating oxidized carbon nanoparticles (MPOCs)” being able to induce the formation of transient pores. However, these NPs quickly bind to the cell membrane, and that binding might induce endocytic uptake even before the pores are formed [80].

### 5.5. Quantum Dots (QDs)

Quantum dots are defined as ellipsoid NPs with a cadmium/selenide core and a zinc sulfide shell having several properties such as small size, surface charge, water solubility, and fluorescence stability that make them good tools for intracellular tracking and cellular imaging. Carboxylic acid-coated QDs are thought to enter the cells by several pathways. Thus, in mesenchymal stem cells (MSCs), the entrance is carried out mainly by CME in absence of serum and by CME and CVME in culture medium [81]. However, QDs are taken up mainly through lipid rafts-CVME in HEK human embryonic kidney epithelial cells and mouse fibroblast NIH-3T3 cells [82]. On the other hand, chemical modifications of these QDs can lead to a change in the uptake pathway. In fact, these same NPs decorated with platelet-derived growth factor (PDGF), switch their uptake pathway to CME [82]. Furthermore, riboflavin modified QDs containing 15 valences entered the cells by CVME, while the ones that contained 70 valences were taken up by CME in human KB cancer cells [83]. In addition, CK2.3 peptide modified QDs enter the cells by CVME in C2C12 immortalized mouse myoblast cell line [84].

### 5.6. Metallic NPs

#### 5.6.1. Iron Oxide NPs (IONPs)

Iron oxide NPs (IONPs) have paramagnetic properties, allowing them to be directed to specific areas using external magnetic fields. IONPs can enter the cells mainly by CVME, at least in immortalized murine macrophages RAW 264.7 line and human ovarian SKOV-3 cancer cells [85]. Moreover, IONPs decoration with dimercaptosuccinate follows CME pathway in rat cerebellar granule neurons and rat oligodendroglial OLN-93 cells [86], rat microglial cells, rat astrocytes, and in MCF-7 breast cancer cells, also with intervention of macropinocytosis in these three last kinds of cells [87]. IONPs decorated with rhodium citrate follows the same CME pathway in MDA-MB231 or MCF-7 human breast adenocarcinoma cell lines [88]. Furthermore, silica coated IONPs enter human cervical carcinoma HeLa cells by CVME while PEGylated ones do it by CVME and CDC42 mediated endocytosis [89].

#### 5.6.2. Gold NPs (AuNPs)

Besides size, shape also plays an important role in Au-NPs uptake. Spherical Au-NPs are taken up by cells better than rod- or bar-shaped Au-NPs being rod-shaped the ones that are more easily extruded from the cell [90]. In addition, rod- and star-shaped Au-NPs seem to enter the cells by CME, with a considerable participation of CVME for the latter in presence of FBS, while in its absence, star-shaped Au-NPs switch uptake to macropinocytosis while rod-shaped Au-NPs are taken up by another independent pathway [91]. Furthermore, 15 nm and 45 nm spherical Au-NPs are endocytosed mainly by CME in the presence of FBS, while in its absence, the NPs are taken up by macropinocytosis due to its aggregation. However, 80 nm ones are taken up by macropinocytosis in absence or presence of FBS, probably because of its larger size [91]. This is in agreement with other studies that indicate that 15 and 30 nm Au-NPs complexed with DNA are taken up by CME [92].

### 5.7. Mesoporous Silica NPs (MSNPs)

Mesoporous silica NPs (MSNPs) are particles based on SiO_2_ that contain a solid framework with a porous structure with good chemical stability and biocompatibility. The pathway followed by MSNPs to enter the cells mainly depends on factors such as size or cell type. Thus, about, 300 nm size silica NPs are taken up through a clathrin- and caveolin-independent process. However, if the NP size is reduced to about 160 nm, NP uptake is carried out by both CME and CVME. Further reductions in size to about 50 nm, lead the NPs to be taken up, apart from CME and CVME, by energy independent processes in human cervical carcinoma HeLa cells [93]. However, some other silica NPs, with different sizes (50, 100, and 150 nm) are also taken up by CME in human cervical carcinoma HeLa cells [94]. On the other hand, shape seems to play an important role as well. Thus, 200 nm rod-shaped SNPs show a larger cellular uptake mediated by CVME than 190 and 90 nm sized spherical SNPs, which enter the cells by CME [95]. In addition, the preferential way of MSNPs entry can hardly vary according to the cell type. Thus, the main way of entrance in NCIH441 human alveolar epithelial cells is flotillin-mediated endocytosis for a wide range of sizes (30-300 nm) [96] while in C2C12 mouse muscle cell line, 50 nm sized MSNPs uptake mainly depends on macropinocytosis and CME [97].

### 5.8. β-Cyclodextrin Based NPs (CDNPs)

β-Cyclodextrins-based NPs (CDs) are composed by a cyclic oligosaccharide with a lipophilic central cavity that can be modified by the addition of different branches. They are widely used in pharmaceutical applications to improve drug bioavailability as carriers [98]. Uptake of CDs seems to be mediated mainly by CME, although some modified CDs such as mono-(6-amino-6-deoxy)-cyclodextrin cannot enter certain cell types such as HeLa human cervical carcinoma cells [99]. However, the addition of amino and guanidine groups to the molecule markedly increases NP uptake by those cells [99]. Moreover, decoration of CDs with moieties targeting specific receptors can change the uptake pathway. For instance, heptamannosylated β-cyclodextrin NPs are selectively internalized by mannose-receptor mediated endocytosis in human breast adenocarcinoma MDA-MD-231 cells [100]. Conjugation of CDs with poly-lysine and hyaluronic acid (HA) are internalized via CD44-mediated endocytosis due to the specific interaction of HA with these CD44 receptors in MHCC-97H and HepG2 human hepatocellular carcinoma cells [101]. Moreover, decoration of CDs with folic acid makes the NPs to be endocytosed by folate receptor-mediated endocytosis in HeLa human cervical carcinoma and A549 human lung carcinoma cells [102].

### 5.9. Micelles

Micelles can be defined as aggregates composed of amphiphilic copolymers which auto-assemble in a liquid, being the lipophilic zone the one that forms the core while the hydrophilic part forms the shell of the micelle. These aggregates are in equilibrium with unimers, which are the free amphiphilic molecules [103].

#### 5.9.1. Gemini Surfactant Micelles

Gemini surfactants are amphiphilic molecules with two head groups and two aliphatic chains, connected by a rigid or a flexible spacer. Most of Gemini surfactants have a common structure [CmH_2_m+1(CH_3_)_2_N+(CH_2_)sN+(CH_3_)_2_C_m_H_2m+1_] 2Br-, or simply m-s-m [104]. The size and the presence of helper lipids (HL) can modify the pathway of Gemini NP entry into the cell. So, several Gemini NPs carrying DNA enter HeLa human cervical carcinoma cells through direct translocation at non-raft domains but if they are attached to HL can be also taken up, in part, by macropinocytosis [104]. As indicated above, size also plays a relevant role in Gemini NP uptake pathway selection. Thus, (14Ser)_2_N_5_/HL complexes with a size of about 200 nm, enter the cells mainly through an energy-independent processes. Increasing NP size to about 550 nm, (16Ser)_2_N_5_-based NPs, switches the uptake pathway to CME. Further increases in size (650–800 nm), i.e., 14-2-14/DNA complexes, leads the NPs to be taken up mainly by macropynocitosis or CVME [105].

#### 5.9.2. Polymeric Micelles

##### Pluronic

Pluronic spherical nonionic micelles are formed by the self-arrangement in water of copolymers like poly (ethylene oxide) (PEO), that becomes hydrophilic and poly (propylene oxide) (PPO), that becomes hydrophobic. Pluronic unimers are capable to enter the cell through CVME while cross-linked micelles can enter the cells by CME [106].

##### Polyethylene Glycol (PEG)

PEG is a polyether widely employed as biomaterial due to its high biocompatibility, low toxicity and immunogenicity and low molecular weight. The uptake mechanism for PEG derived micelles is dependent on CME and CVME, being that this last process is slightly more implicated. These are the pathways for 20–30 nm sized electropositive PEG-*co*-poly(ε-caprolactone) (PEG-PCL) and PEG-(distearoyl-snglycero-3-phosphoethanolaminen) (PEG-DSPE) micelles in MDCK dog kidney epithelial cells [107] and for 30 nm sized methoxyPEG-PLGA (mPEG-PLGA) micelles in Calu-3 and NCI-H441 human lung adenocarcinoma cells [108]. Furthermore, for electronegative PEG-polylactic acid (PEG-PLA) 45 nm sized micelles, the uptake is dependent of CVME in MDCK dog kidney epithelial cells [109], the same uptake pathway that neutral charged PEG-D-tocopheryl succinate 15 nm sized micelles in A549 human lung carcinoma cells [110].

##### Hyaluronic Acid (HA)

HA is a linear polysaccharide highly biocompatible, biodegradable, mucoadhesive, and viscoelastic employed in several biomedical applications. Neutral 130 nm sized micelles with oleyl-hyaluronan (HAC18:1) and hexyl-hyaluronan (HAC6) covalently linked enter the cells by CME and macropinocytosis in HaCaT human immortalized keratinocites [111]. However, as in other particle kinds, functionalization of these kind of micelles can lead to a change in the pathway, as happens with electronegative 160 nm sized HA-octadecylamine conjugate functionalized with N-acetylcysteine, in which the way of entrance to the cell is CVME apart from CME in Caco-2 and HT29 human colon cancer cells [112].

## 6. Conclusions

The use of NPs as carriers for different therapeutic compounds (small-drugs, siRNA, etc.) has become very common because of the multiple opportunities provided by the chemical nature of the NPs, including facile modification of their surface terminal groups to allow them to be directed to the target cells. To properly deliver their therapeutic cargo, the NPs must, first, be taken up by the target cells. The internalization pathway followed by the NP and its cargo is very relevant since it can help to modify its intracellular fate. Most of the NPs are taken up by pinocytosis, mainly through receptor-mediated endocytosis.

There are different NP properties that might play a key role in both NP extent of uptake and in the endocytic pathway followed by the NP and its cargo. Some of those physico-chemical characteristics are: size, charge, shape, and rigidity. All these factors can be considered as critical parameters to be taken into account when designing NPs since a few rules can be derived from the work already performed with different NPs. So, to facilitate cellular uptake of the NPs and their cargos, a smaller size with positive charge and a rigid structure seem to be the most favorable properties to facilitate cellular uptake. These general rules seem to facilitate an in-silico design of the NPs and should be considered when designing a specific NP to deliver therapeutic cargos to different cell types. A summary of the main endocytic pathways and the effect of physico-chemical characteristics on uptake mechanisms can be found in Table 1.

However, there are other players coming into the game that blur this apparently clear picture. The first is the different chemical nature of the diverse NP types that direct them towards different endocytosis pathways. Moreover, even for the same type of NP, modifications in the surface chemical groups or decoration with different ligands to increase cellular targeting can markedly vary the cellular entry pathway, making it very difficult to make predictions about the rate and extent of NP entry and cargo delivery for several given NPs with distinct chemical nature. On top of that, for the same chemical entity, different cell types take up the same NPs following different routes. This fact might be related to the different lipid, protein, and sugar composition of the external part of the cell membrane in cells from different origins. This further complicates establishing common rules to be followed to design efficient NPs.

The fact that different endocytic routes are taken by the same NP to enter different cell lines stresses that, for a NP to be aimed to be studied in animal models of disease or to enter the clinical setting, it would be desirable that the approach to explore the endocytic pathway followed by the NP will be studied in primary target cell (neurons, astrocytes, macrophages, hepatocytes, etc.) cultures and not in cell lines to be certain about the endocytic pathway that likely would be followed by the NP in the primary cell target in either the animal of the human body.

In the future, NP design for a successful delivery into the cell should be based in a better knowledge of the functional groups that are relevant for NP uptake, as well as the preferential pathways activated by the binding of targeting groups to different receptors used as targets for selective delivery of NPs and cell cargos to certain cell types. However, while that new knowledge is available, trial and error testing of the newly designed and decorated NPs seems to be the only available approach for studying the pathways to be followed by a NP to be taken up by the cells.

## Figures and Tables

**Table 1:** Main endocytosis pathways for every nanoparticle (NP) kind described in this review article, considering factors as size, charge, or shape.

NP Type		Main Endocytic Pathway	Size/Length	Charge	Shape	Reference(s)
Natural polymers	CS	CME	15–250 nm	Positive	Ellipsoidal and spherical	[25,26]
	Albumin	CME	140 nm	Positive	Spherical	[31]
		CME	150 nm	Negative	Spherical	[30]
		CVME	120 nm	Negative	Spherical	[32]
	Alginate	CME	50–120 nm	Negative	Spherical	[33]
		CVME	420 nm	Negative	Spherical	[33]
		Macropinocytosis	730 nm	Negative	Spherical	[33]
Synthetic polymers	Polystyrene	CME and passive diffusion	40–150 nm	Negative	Not specified	[34,35,36,37]
	PLGA	CME	80 nm	Positive	Not specified	[38]
		Weak entrance CME and CVME independent	80 nm	Negative	Not specified	[38]
	PEI	CME and CVME	100–130 nm (25 kDa)	Positive	Branched	[43,44,45,46]
		CME	25 kDa	Positive	Linear	[43]
Dendrimers	PAMAM -NH2	CME	G4 (5–150 nm)	Positive	Branched	[35,40,41,42]
		CME and CVME	G2	Positive	Branched	[43,44]
	PAMAM -OH	CVME	G4	Negative	Branched	[53]
	PAMAM -COOH	CVME	G3.5	Negative	Branched	[53]
		CME	G1.5	Negative	Branched	[54]
	PAMAM	CME and CVME independent	G4	Neutral	Branched	[53]
Lipids		CME and macropinocytosis	100–150 nm	Positive	Spherical	[59,60,66]
	Liposomes	CME and macropinocytosis	100 nm	Negative	Spherical	[66]
		CME and CVME	100 nm	Neutral	Spherical	[66]
	SLNs	CME	110–160 nm	Positive	Not specified	[67]
		CME, CVME and macropinocytosis	85–90 nm	Negative	Not specified	[68]
Carbon based	SWCNTs	Macropinocytosis and non-specific interactions	195–630 nm	Negative	Cylindrical	[72,73]
		Passive diffusion	50 nm	Negative	Cylindrical	[74]
	MWCNTs	CME and CVME	10 µm	Negative	Cylindrical	[75]
	Fullerenes	Passive diffusion	1 nm (55 nm aggregates)	Negative	Icosaedral	[78]
	Carbon oxide NPs	Unspecific interactions	38 nm (225 nm aggregates)	Negative	Irregular	[80]
QDs		CVME and CME	10–50 nm	Negative	Ellipsoidal	[81,82]
Metallic	IONPS	CVME	15–50 nm	Negative	Not specified	[85]
		CME (Macropinocytosis in absence of FBS)	15–45 nm	Negative	Spherical	[91,92]
	AuNPs	Macropinocytosis	80 nm	Negative	Spherical	[91]
		CME and CVME (Macropinocytosis in absence of FBS)	15 nm	Negative	Star	[91]
		CME (CME and CVME independent way in absence of FBS)	33 × 10 nm	Negative	Rod	[91]
MSNPs		CME and CVME independent	300 nm	Negative	Not specified	[93]
		RME, macropinocytosis and simple diffusion	50–300 nm	Negative	Not specified	[93,94,97]
		CVME	200 nm	Negative	Rod	[95]
		CME	90–190 nm	Negative	Spherical	[95]
CDNPs		CME	40–140 nm	Positive	Not specified	[99]
Micelles	Gemini surfactants (14-2-14, 16-2-16, 12-2-12, 12-5-12, 12-10-12)	Direct translocation	3 µm (1–6 µm)	Positive	Spherical	[104]
	Gemini surfactants with HL (14-2-14, 16-2-16, 12-2-12, 12-5-12, 12-10-12)	Macropinocytosis	3 µm (1–6 µm)	Negative	Spherical	[104]
	Gemini surfactant (14Ser)2N5/ DNA/HL	Energy independent processes	200 nm	Negative	Spherical	[105]
	Gemini surfactant (16Ser)_2_N_5_/DNA	CME	550 nm	Positive	Spherical	[105]
	Gemini surfactant 14-2-14/DNA (with or without HL)	Macropinocytosis and CVME	555–800 nm	Positive	Spherical	[105]
	Pluronic	CVME	2–5 nm	Neutral	Unimers	[106]
		CME	15-50 nm	Neutral	Cross-linked micelles (spherical)	[106]
	PEG-PCL PEG-DSPE	CME and CVME	20–30 nm	Positive	Spherical	[107]
		CME and CVME	20–30 nm	Positive	Spherical	[107]
	mPEG-PLGA	CME and CVME	30 nm	Not specified	Spherical	[108]
	PEG-PLA	CVME	45 nm	Negative	Spherical	[109]
	PEG-D-tocopheryl succinate	CVME	15 nm	Neutral	Spherical	[110]
	HA	CME and macropinocytosis	130 nm	Neutral	Spherical	[111]

Abbreviations: NP = nanoparticle, CS = chitosan, PLGA = poly(lactic-co-glycolic) acid, PEI = polyethylenimine, PAMAM = polyamidoamine, SLNs = solid lipid NPs, SWCNTs = single-walled carbon NPs, MWCNTs = multi-walled carbon NPs, QDs = quantum dots, IONPs = iron oxide NPs, AuNPs = gold NPs, FBS = fetal bovine serum, MSNPs = mesoporous silica NPs, CDNPs = β-cyclodextrin-based nanoparticles, HL = helper lipid, CME = clathrin-mediated endocytosis, CVME = caveolin-mediated endocytosis, PEG-PCL = PEG-co-poly(ε-caprolactone), PEG-DSPE = PEG-(distearoyl-snglycero-3-phosphoethanolaminen), mPEG-PLGA = methoxyPEG-PLGA, PEG-PLA = PEG-polylactic acid, HA = hyaluronic acid.
